# The complete mitochondrial genome of *Epicauta ruficeps* (Coleoptera: Meloidae)

**DOI:** 10.1080/23802359.2020.1763213

**Published:** 2020-05-13

**Authors:** Xiaohong Han, Yechen Li, Ciding Lu, Guanghong Liang, Feiping Zhang

**Affiliations:** a College of Forestry, Fujian Agriculture and Forestry University, Fuzhou, China; b Key Laboratory of Integrated Pest Management in Ecological Forests, Fujian Province University, Fujian Agriculture and Forestry University, Fuzhou, China

**Keywords:** Complete mitochondria genome, *Epicauta ruficeps*, Meloidae, phylogenetic analysis

## Abstract

*Epicauta ruficeps* is widely distributed in China and some countries in Southeast Asia, and plays an important role in medicine and biological control. The complete mitochondria genome of *E. ruficeps* was 15,813 bp in length, with 37 genes, including 13 PCGs, 22 tRNA genes (tRNAs), and two rRNA genes (rRNAs). The positions and sequences of genes were consistent with those of known Meloidae species. The nucleotide composition was highly A + T biased, accounting for ∼65% of the whole mitogenome. The complete mitogenome of *E. ruficeps* would help understand Meloidae evolution.

Meloidae insects’ unique life cycle, larval parasitism, adult gregariousness, chemical defense, drought tolerance and other interesting biological characteristics, make this group classification and other studies receive extensive attention (Wang et al. 2010). *Epicauta ruficeps* (Coleoptera: Meloidae) is widely distributed in China and some countries in Southeast Asia, and plays an important role in medicine and biological control (Wang et al. 2010). The adult is able to secrete cantharidin which has obvious anticancer and insecticidal properties (Moed et al. 2001; Fang et al. 2001; Li et al. 2009). In this study, we reported the complete mitochondrial genome of *E. ruficeps* and phylogenetic analysis for the first time (GenBank accession No: MN913338). The sample of *E. ruficeps* was collected from Sanming (26°13′N, 117°36′E), Fujian Province, China, in June 2019, and the specimens are deposited in Key Laboratory of Integrated Pest Management in Ecological Forests, Fujian Agriculture and Forestry University, Fuzhou, China (voucher no. ER-201906). DNA materials were extracted from tissues using TruSeq DNA sample Preparation kit (Vanzyme, China). The complete mitochondrial genomes were obtained through Illumina Hiseq 2500.

The complete mitogenome of *E. ruficeps* was circular in shape and 15,813 bp in length with 35.36% GC content. In total, 14,496 genes were annotated, containing 13 protein-coding genes (PCGs), 22 transfer RNA (tRNAs), and two ribosomal RNA (rRNAs), and 1317 nucleotides were non-coding DNA (D-loop region). The composition and arrangement of the mitogenome was comparable to the case of other Meloidae (Jie et al. 2016; Wu et al. 2018). The nucleotide composition was highly A + T biased, the A + T content of PCGs, tRNAs, and rRNAs was 64.96%, 71.62%, and 71.47%, respectively. The first genes started from *trnM* in the same direction with other Meloidae insects (Jie et al. 2016; Wu et al. 2018).

Phylogenetic analysis was constituted by 13 PCGs sequence of *E. ruficeps* the with the other 9 species of Meloidae, and we chose *Tribolium castaneum* as outgroup. The phylogenetic tree was built by using the Neighbor-Joining (NJ) method with 1000 bootstrap replicates through MEGA 7.0 (Sudhir et al. 2016). The result showed that *E. ruficeps* was closely related to *E. aptera*, they evolved earlier than other species of *Epicauta*, and the phylogenetic relationship among Meloidae was (((*Epicauta*) + *Lytta*) + *Hycleus*) (Figure 1). In conclusion, the complete mitogenome of *E. ruficeps* reported in this study provided important information for the phylogeny and evolution analysis of Meloidae.

**Figure 1.**
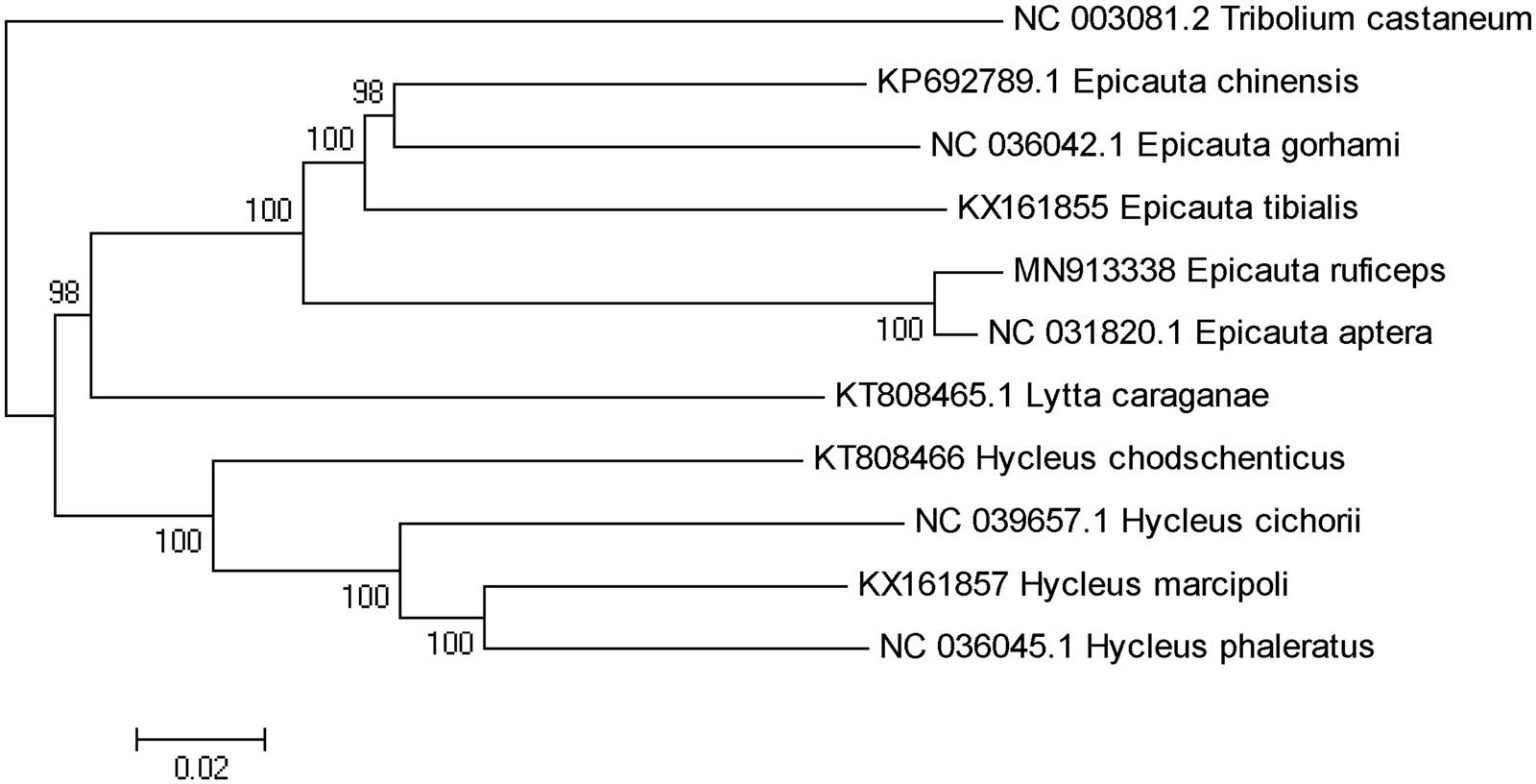
Neighbor-joining tree based on 13 PCGs of 10 Meloidae by using MEGA 7.0 (bootstrap values based on 1000 replicates).

## Data Availability

The data that support the findings of this study are openly available in “NCBI” at https://www.ncbi.nlm.nih.gov/, reference number MN913338.
